# Health workers’ motivation was significantly higher in private hospitals than public hospitals influenced by intrinsic and extrinsic factors in Northwest Ethiopia

**DOI:** 10.3389/fpubh.2025.1452229

**Published:** 2025-05-12

**Authors:** Gebremariam Ayichew, Desta Debalkie Atnafu, Mohammed Hussein, Ayenew Takele Alemu

**Affiliations:** ^1^Department of Health System Management and Health Economics, School of Public Health, Bahir Dar University, Bahir Dar, Ethiopia; ^2^International Center for Evidence in Disability, London School of Hygiene & Tropical Medicine, London, United Kingdom; ^3^Department of Public Health, College of Medicine and Health Sciences, Injibara University, Injibara, Ethiopia

**Keywords:** motivation, public and private hospitals, health workers, Bahir Dar City, Ethiopia

## Abstract

**Background:**

Motivation of health workers is crucial for providing high-quality healthcare services and improving the performance of health facilities. However, less attention has been given to this aspect of workplace climate in hospital settings, and there is scant evidence on the level of health workers’ motivation on healthcare delivery. Therefore, this study aimed to assess the motivation of health workers and its determinants at public and private hospitals in Bahir Dar City, Northwest Ethiopia.

**Methods:**

A facility-based comparative cross-sectional study was conducted from November 3rd to December 4th, 2021. A simple random sampling technique was used to select 472 study participants. Motivational status was measured using the mean of 14 items on a Likert scale. Descriptive statistics were computed to present results using tables and figures. Bi-variable and multivariable logistic regressions were performed to identify factors associated with motivational status. Strength of association was measured using adjusted odds ratios with the corresponding 95% confidence intervals and statistical significance was declared at *p*-values less than 0.05.

**Results:**

A total of 458 health workers participated in our study the health workers’ motivational status was found to be 56.3% (95% CI: 52–60.7%). A significant difference in motivational status was observed between private (62.3%) and public hospitals (50.4%) (χ^2^ = 6.532, *p* = 0.011). Working in private hospitals (AOR = 1.52; 95% CI: 1.03–2.23), good collegial relationships (AOR = 1.61; 95% CI: 1.1–2.32), job satisfaction (AOR = 1.49; 95% CI: 1.02–2.20), a favorable work environment (AOR = 1.56; 95% CI: 1.06–2.30), and educational status (AOR = 0.4; 95% CI: 0.17–0.94) were significantly associated with higher health workers’ motivational status.

**Conclusion:**

The motivational status of health workers was significantly higher in private hospitals than in public hospitals. The proportion of motivated health workers was low, which poses challenges in maintaining a regulated health workforce within the health system. Working in private hospitals, job satisfaction, working environment, higher educational status, and collegial relationships were predictors of motivational status. Therefore, public hospitals should promptly implement both intrinsic and extrinsic motivational strategies.

## Introduction

In the workplace, motivation is defined as an individual’s willingness to exert and sustain effort. It encompasses the readiness to work productively toward achieving organizational goals. Motivated individuals are energized to overcome challenges and enhance their accomplishments. Therefore, motivation plays a crucial role in maximizing organizational performance (1–3). According to Herzberg’s two-factor theory, motivation can be categorized into two types: extrinsic and intrinsic motivation. Intrinsic motivation originates from within individual workers and is often associated with factors such as job satisfaction, recognition, achievement, and advancement. Conversely, extrinsic motivation is linked to external factors such as incentives, the working environment, and management practices (4–6). Intrinsic and extrinsic motivators are not entirely independent of each other; rather, they exhibit an inverse relationship. Intrinsic motivators tend to enhance motivation when they are present, while extrinsic motivators have the potential to diminish motivation when they are lacking (7).

Motivation in healthcare delivery positively impacts quality and patient safety (8). In a decentralized governing system, a motivated workforce is essential for developing a well-functioning health system (9). Resources availability and professional competencies are not sufficient to attain health system goals without adequate motivational packages (10–13). Healthcare delivery is a time-consuming and complicated system. Highly motivated health workers are required to meet the patients’ desired expectations and competing clinical issues (14). In Ethiopia, the Ministry of Health has introduced different reforms and approaches such as business process re-engineering, healthcare financing, and health information systems to improve the quality of healthcare by adhering to a strong management system (15, 16). However, the health system is currently experiencing high turnover rates, with trained health professionals, particularly in the public sector, migrating and leaving their positions (17–19).

In Ethiopia, health workers across various disciplines in hospital settings were not adequately motivated. Only a quarter (25.1%) of health workers in Jimma Comprehensive Specialized Hospital reported being motivated (14). In central Ethiopia, it was reported that the level of motivation among health workers accounted for 63.63% (20). In Gedeo zone, Ethiopia, it was found that 50% of health workers were not motivated. Only 19.5% of health workers working in health centers were highly motivated (21). In public hospitals of west Amhara and Debre Markos Comprehensive Specialized Hospital, the level of motivation among health workers reached 58.6 and 20.4%, respectively (16, 22). Studies that were conducted abroad to Ethiopia revealed that health workers motivation was 42.7% in Cameroon (23), 75.55% in Kenya (24),

Recognizing and addressing the demotivating factors is a central dimension of managing employees’ motivation (25, 26). Herzberg’s two-factor theory is relevant to human resource management in the health sector. It identifies company policy and administration, supervision, interpersonal relations, working conditions, and salary as hygiene factors of motivation (27). Workers are motivated not only by extrinsic rewards but also by the desire to fulfill their psychological needs, such as autonomy, competence, and relatedness (28). Fort and Voltero in Armenia revealed that performance improvement regarding maternal healthcare was enhanced following incentives to the providers in the form of recognition, in-kind contributions, community respect, and assistance with services (29). In Uganda, it was evidenced that unfavorable working conditions, low remuneration, and lack of recognition and promotion were contributing to the inadequate level of health workers’ motivation (30). Good management, supportive supervision, and positive relationships with colleagues were identified as factors that enhance health workers’ motivation (31–34). It was also evidenced that health workers were motivated to perform better when provided with training opportunities, promotions, conducive working environment, effective leadership and management, competitive salaries, and manageable workloads (35–37). The motivation level of health workers was significantly related to factors such as their age, educational level, work experience, administrative positions, financial benefits, and communication (38). Job satisfaction, training opportunities and advancement in professional carriers are among positively contributing factors for higher level of health workers motivation (22, 39, 40).

Strategic human resource management has been taken into account as important tool to motivate and maintain healthcare professionals (41). The poor motivation of health workers contributes to the weakness of many nations’ health systems. However, adequate attention has not been provided for this significant issue of healthcare delivery system. While some studies have attempted to examine health workers’ motivation in Africa, their measurements were not aligned with the contexts of developing nations (20, 42, 43). To the best of the authors’ knowledge, few studies on health workers’ motivation have been conducted in Ethiopia. However, evidence is very limited in the study area. Thus, this study aimed to determine the level of health workers’ motivation and its associated factors at public and private hospitals in Bahir Dar City. our study was also intended to compare health workers’ motivation between public and private hospitals.

## Materials and methods

### Study design and setting

A facility-based comparative cross-sectional study was conducted from November 3rd to December 4th, 2021, in Bahir Dar City, located 565 kilometers away from Addis Ababa, the capital City of Ethiopia. Both private and public hospitals were included as study institutions. According to the Municipality report (2021), Bahir Dar City has a total population of 345,084 residents (44). There are three public hospitals (Felege-Hiwot Comprehensive Specialized Hospital, Tibebe-Gion Comprehensive Specialized Hospital, and Addis Alem Primary Hospital) and four private hospitals (Dream Care, Gamby, Adinas, and Afilas General Hospitals) in the City. The hospitals are currently servicing more than 5 million populations in outpatient, emergency, inpatient, and maternity units for essential healthcare including medical, surgical, pediatrics, orthopedics, ophthalmic, and obstetric-gynecologic services. These hospitals are currently employed by 1820 health workers.

### Study participants and eligibility criteria

The study participants were healthcare professionals of all disciplines including physicians, nurses, midwives, pharmacists, anesthetists, health officers, physiotherapists, and laboratory technologists who were working in public and private hospitals in Bahir Dar City administration. All healthcare workers employed in these hospitals constituted the source population. We included all full-time working health professionals with a work experience of 6 months or more. However, we excluded healthcare professionals who were newly employed and had maternal or annual leave during the data collection period.

### Sample size determination

The sample size for objective one was calculated using the double-population proportion (public hospitals and private hospitals) formula. We considered a 95% confidence interval, a 5% margin of error, a 58.3% proportion (P1) for public hospitals taken from a recent study in Ethiopia (39), a 50% proportion (P2) for private hospitals, as there was no previous study in Ethiopia, and 10% for non-response rate. Accordingly, a sample size of 561 was estimated. For objective two, the sample size was estimated considering a 95% confidence interval, 80% power, and a 10% non-response rate, based on significantly associated factors from previous studies (14, 39). The maximum sample size was 374. Since the largest sample size was taken for objective one, the final sample size was determined to be 561 (281 for each of public and private hospitals) using a 1:1 ratio.

### Sampling procedure

The study participants were selected from all three public and four private hospitals in the City administration. The study populations were homogeneous since all were health professionals, and the study units were grouped by their working facility. The required sample size for each public and private hospital was allocated proportionally based on the number of health workers: 151 health workers in Felege-Hiwot Comprehensive specialized hospital, 100 in Tibebe-Gion Comprehensive specialized hospital, 28 in Addis Alem Primary hospital, 69 in Dream Care, 100 in Gamby, 73 in Adinas, and 37 in Afilas general private hospitals. The study participants from each hospital were selected using a simple random sampling technique.

### Study variables

#### Dependent variable

The health workers’ motivational status was outcome variable of our study.

#### Independent variables

Socio-demographic variables include age, sex, marital status, religion, profession, workplace, work experience, monthly salary, educational status, and managerial position. Intrinsic factors comprised of achievement, recognition, advancement, and responsibility, while extrinsic factors include collegial relationships, working environment, remuneration, justice and fairness, and resource availability. Additionally, job satisfaction, performance appraisal, salary/career structure, duty payment, working hour schedule, and transportation were considered.

### Operational definitions

Health workers in this study refer to healthcare providers who were full-time employees of public or private hospitals, including physicians, nurses, midwives, pharmacists, laboratory technologists, anesthetists, health officers, and others such as physiotherapists, radiographers, and environmental health specialists. Motivational status was measured using 14 items of general motivation questions. Health workers were asked to provide their responses using a 5-point Likert scale ranging from 1 (strongly disagree) to 5 (strongly agree). The health workers’ motivation was dichotomized into “motivated” and “not motivated” based on the calculated cut-off point (mean = 3.2). If the mean score of the respondent equaled or exceeded the overall mean, we considered the health worker as motivated. Conversely, if it was below the overall mean, we considered the health worker as not motivated (14, 45).

### Data collection tools and procedures

A self-administered structured questionnaire tool was adopted from similar studies conducted in Ethiopia and Zambia (14, 39, 46) and used to collect the data. The questionnaire was prepared in English and comprised of sections on socio-demographic characteristics, factors influencing motivation, and motivational status. Data collection was conducted by two trained diploma nurses, with supervision provided by a BSc nurse.

### Data quality control

A validated data collection tool was adopted and pre-tested using 5% of the sample size outside of the study hospitals. Internal consistency (reliability) of Likert Scale questions was checked by calculated Cronbach’s alpha value which was 0.86 in our study. One day of training for data collectors and supervisors was provided regarding instrument contents and data collection procedures. Data completeness and consistency were checked daily by the authors and supervisor.

### Data analysis and management

Epi-Data version 3.1 was used for data entry and cleaning. The data were then exported to SPSS version 23 for statistical analysis. Descriptive statistics were employed to present study results through tables and figures. The Chi-square (χ^2^) test was computed to assess the difference in motivational status between public and private hospitals. Binary logistic regression was used to examine the association between dependent and independent variables. Explanatory variables with *p*-values less than 0.25 in the bivariable analysis were considered as candidates for multivariable analysis. Significant associations were identified and interpreted using an adjusted odds ratio with a 95% CI at *p*-value less than 0.05. The model’s fitness was checked using the Hosmer-Lemeshow goodness-of-fit test (*p* = 0.568). Multicollinearity was assessed using the variance inflation factor (VIF).

## Results

### Socio-demographic characteristics

A total of 458 health workers, 230 from public hospitals and 228 from private hospitals, responded to the research questions, resulting in a response rate of 81.5%. The mean age of the respondents was 31.38 ± 5.70 (SD) years. The majority of health workers (60%) were male. Approximately, two-thirds of the participants (66.6%) were married. Regarding religion, the majority (85.8%) were identified as Orthodox Christian followers. Nurses constituted the largest group of health workers followed by physicians, accounting for 33 and 19.9%, respectively. More than half of the respondents (54.6%) held a bachelor’s degree as their highest qualification. A large proportion of the participants (43.9%) had 1–5 years of work experience. Overall, the average monthly salary was 9351.70 ± 8084.39 (SD) in Ethiopian birr. Sex, age, marital status, work experience, educational status, and monthly salary characteristics showed significant variation between public and private hospital employees (Table 1).

**Table 1:** Socio-demographic characteristics of health workers at hospitals in Bahir Dar City, Northwest Ethiopia, 2021.

Characteristic	Category	Health institutions (*n* = 458)		χ^2^ (*p*-value)
		Public-230	Private-228	
Sex	Male	74(32.2)	109(47.8)	11.6(0.001)
	Female	156(67.8)	119(52.2)	
Age	≤24	7(3)	20(8.8)	16.3(0.003)
	25–29	68(29.6)	89(39)	
	30–34	95(41.3)	80(35.1)	
	35–39	34(14.8)	17(7.5)	
	>40	26(11.3)	22(9.6)	
Religion	Orthodox	201(87.4)	192(84.2)	0.3(0.86)
	Protestant	15(6.5)	24(10.5)	
	Muslim	14(6.1)	12(5.3)	
Marital status	Single	63(27.4)	84(36.8)	6.45(0.04)
	Married	154(67)	138(60.5)	
	Others ^a^	13(5.7)	6(2.6)	
Profession	Midwifery	31(13.5)	31(13.5)	3.85(0.57)
	Nurse	81(35.2)	70(30.7)	
	Physician	47(20.4)	44(19.3)	
	Laboratory	26(11.3)	24(10.5)	
	Pharmacy	22(9.6)	23(10.1)	
	Others ^b^	23(10)	36(15.8)	
Work experience	<1 year	2(0.9)	31(13.5)	31(0.000)
	1–5 years	102(44.3)	99(43.4)	
	6-10 years	73(31.7)	66(28.9)	
	>10 years	53(23)	32(14)	
Educational status	Diploma	20(7.8)	71(31.1)	43.4(0.000)
	Degree	149(64.8)	101(44.3)	
	Masters/above	61(26.5)	56(24.6)	
Monthly salary in ETB	<6,000	25(10.9)	96(42.1)	62.2(0.000)
	6,001–10,000	148(64.3)	80(35.1)	
	>10,000	57(24.8)	52(22.8)	
Managerial position	Yes	19(8.3)	23(10.1)	
	No	211(91.7)	205(89.9)	

^a^: divorced or widowed, others. ^b^: Anesthetist, health officer, radiologists, environmental, physiotherapy, χ^2^: Chi-Square.

### Extrinsic factors of motivation

Eighty-two (35.7%) and 118 (51.8%) health workers employed in public and private hospitals, respectively, perceived the working environment as favorable. More than half of public (57%) and private (56.1%) health workers reported having poor and good collegial relationships, respectively. The majority (65.2%) of public health workers indicated inadequate drug, supply, and equipment availability in their hospitals, while 52.6% of private health workers reported adequate supply and drug availability. Regarding extrinsic factors, significant variations were observed between public and private hospital employees, particularly in interpersonal relationships and remuneration factors (Table 2).

**Table 2:** Perceptions of extrinsic motivational factors among health workers at hospitals in Bahir Dar City, Northwest Ethiopia, 2021.

Variables	Category	Health institutions (*n* = 458)		χ^2^ (*P*-value)
		Public	Private	
Working environment	Favorable	82(35.7)	118(51.8)	0.01(0.92)
	Not favorable	148(64.3)	110(48.2)	
Interpersonal relationship	Good	99(43)	128(56.1)	7.86(0.005)
	Poor	131(57)	100(43.9)	
Justice and fairness	Fair	114(49.6)	123(53.9)	0.88(0.35)
	Unfair	116(50.4)	105(46.1)	
Supply, drugs, and equipment	Adequate	80(34.8)	120(52.6)	0.137(0.711)
	Inadequate	150(65.2)	108(47.4)	
Remuneration	Remunerated	83(36.1)	105(46.1)	4.7(0.03)
	Not remunerated	147(63.9)	123(53.9)	

χ^2^: Chi-square.

### Intrinsic factors of motivation

Ninety-seven (44.3%) respondents in public hospitals and 107 (46.9%) in private hospitals, believed that they had achieved. Only 105 (45.7%) participants from public hospitals and 97 (42.5%) from private hospitals explained that they had advanced in their careers. In private hospitals, the majority of respondents (65.4%) perceived that they had been recognized for their better performance, while in public hospitals, only 32.2% of the respondents explained that they were provided recognition. A significant difference was observed in the recognition factor between public and private hospital employees (Table 3).

**Table 3:** Health workers’ perception of intrinsic factors of motivation at hospitals in Bahir Dar City, Northwest, Ethiopia, 2021.

Variables	Category	Health institutions		χ^2^ (*P*-value)
		Public	Private	
Achievement	Achieved	102(44.3)	107(46.9)	0.31(0.579)
	Not achieved	128(55.7)	121(53.1)	
Advancement	Advanced	105(45.7)	97(42.5)	0.449(0.503)
	Not advanced	125(54.3)	131(57.5)	
Recognition	Recognized	74(32.2)	149(65.4)	50.446(0.0001)
	Not recognized	156(67.8)	79(34.6)	

### Job satisfaction

**Figure 1:**
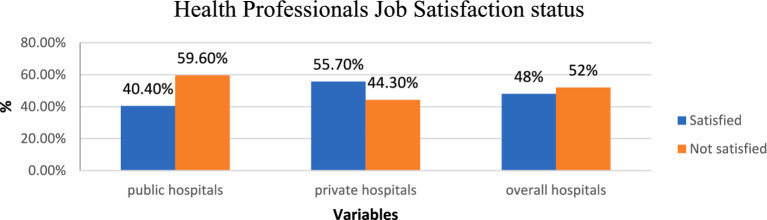
Job satisfaction among healthcare professionals in public and private hospitals in Bahir Dar City, 2021.

The overall job satisfaction level of healthcare workers was 52%. There was a statistically significant difference in job satisfaction based on employment status ownership (χ^2^ = 10.69, *p* < 0.001). Job satisfaction among private hospital workers (55.7%) was higher than among public hospital workers (40.4%) (Figure 1). Better recognition in private hospitals increased employees’ job satisfaction, while improved compensation and staff relationships in private hospitals decreased employees’ job dissatisfaction.

### The motivational status of health workers

**Figure 2:**
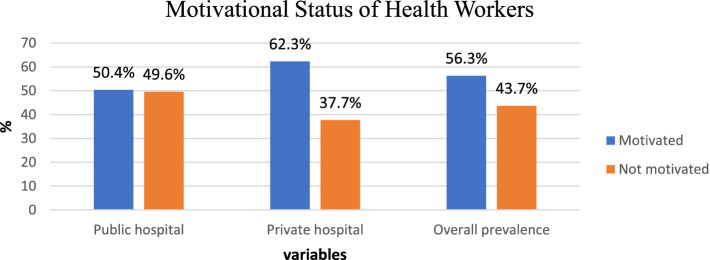
Motivation status among public and private hospitals in Bahir Dar City, 2021.

The proportion of motivated health workers was revealed to be 56.3% (95% CI: 52–60.7). It was 50.4 and 62.3% for public and private hospital employees, respectively. The study showed a significant difference in the health workers’ motivational status between public and private hospitals in Bahir Dar City (χ^2^ = 6.53, *p* = 0.011) (Figure 2).

### Factors associated with the health workers’ motivational status

The current study demonstrated that there was a strong relationship between type of hospital ownership and the health workers’ motivational status. Health workers who were working in private hospitals were 1.52 times more likely to be motivated when compared to those who were working in public hospitals (AOR = 1.52, 95% CI: 1.03–2.23). The odds of motivational status of respondents with good collegial relationship were 1.61 times more likely to be motivated than their counter parts (AOR = 1.61 at 95%CI = 1.10–2.36). Health workers who were satisfied in their jobs were 1.49 times more likely to be motivated than those who were not satisfied (AOR = 1.49, 95% CI: 1.02–2.20). The motivational status increased by 1.56 times among respondents who were working in a favorable working environment compared to those who were working in unfavorable conditions (AOR = 1.56, 95% CI: 1.06–2.30). Health workers who hold a Master’s degree showed a 60% reduction in the level of motivational status compared to those with a diploma (AOR = 0.40, 95% CI: 0.17–0.94). Our study revealed that extrinsic factors were more pronounced in hospitals of Bahir Dar City (Table 4).

**Table 4:** Factors associated with the motivational status among health workers at hospitals in Bahir Dar City, Northwest, Ethiopia, 2021.

Variables	Category	Motivational status		COR(95% CI)	AOR(95% CI)
		Motivated	Not-motivated		
Hospital ownership	Public	116(50.4)	114(49.6)	1	1
	Private	142(62.3)	86(37.7)	1.62(1.12, 2.35)	1.52(1.03, 2.23)*
Achievement	Achieved	131(62.7)	78(37.3)	1.61(1.11, 2.35)	1.36(0.92, 2.01)
	Not achieved	127(51.0)	122(49.0)	1	1
Supply, drugs and equipment	Adequate	133(60.7)	86(39.3)	1.41(0.97, 2.05)	1.03(0.68, 1.56)
	Inadequate	125(52.3)	114(47.7)	1	1
Working environment	Favorable unfavorable	127(63.5) 131(50.8)	73(36.5) 138(49.2)	1.69(1.16, 2.46)	1.56(1.06, 2.30)*
Job satisfaction	Satisfied	139(63.2)	81(36.8)	1.72(1.18, 2.50)	1.49(1.02, 2.20)*
	Not satisfied	119(50)	119(50)	1	1
Interpersonal relationship	Good	141(63.8)	80(36.2)	1.81(1.24, 2.63)	1.61(1.10, 2.36)*
	Poor	117(49.4)	120(50.6)	1	1
Remuneration	Remunerated	113(60.1)	75(39.9)	1.30(0.89, 1.89)	0.98(0.64, 1.52)
	Not-remunerated	145(53.7)	125(46.3)	1	1
Educational level	Diploma	58(63.7)	33(36.3)	1	1
	Degree	143(57.2)	107(42.8)	0.76(0.46, 1.25)	0.89(0.52, 1.52)
	Master	13(37.2)	22(62.9)	0.34(0.15, 0.75)	0.40(0.17, 0.94)*
	GP/Specialist	44(53.7)	38(46.3)	0.66(0.36, 1.21)	0.74(0.40, 1.40)
Monthly salary (ETB)	≤6,000	79(65.3)	42(34.7)	1	1
	6,001–10,000	121(53.1)	107(46.9)	0.60(0.38, 0.95)	0.72(0.41, 1.28)
	≥10,000	58(53.2)	51(46.8)	0.61(0.36, 1.03)	0.86(0.37, 1.98)
Managerial position	Yes	20(47.6)	22(52.4)	0.68(0.36, 1.28)	0.67(0.34, 1.32)
	No	238(57.2)	178(42.8)	1	1

*p < 0.05; 1 = reference category.

## Discussion

Enhancing the quality of healthcare services, lowering burnout, and guaranteeing patient satisfaction all depend on motivating healthcare professionals. Motivational techniques should take into account social, professional, and economical aspects in Ethiopian healthcare systems, as well as those in other countries (26). Motivated health workers are energetic to exert their creative potentials in healthcare delivery and can apply their knowledge and skills in establishing goal oriented health system (39). The present study was conducted to determine the level of health workers motivation and to identify associated factors in both private and public hospitals of Bahir Dar City.

Our study revealed that the overall motivational status among health workers of hospitals in Bahir Dar City was 56.3%. This finding is almost similar to other findings from Gedeo zone (Southern Ethiopia), Amhara (Northwest Ethiopia), Arsi zone (Oromia region), and Jimma town (Southwest Ethiopia) which reported 54.8, 58.6, 58.3, and 54.5% motivational status, respectively (16, 21, 39, 45). The possible explanation for these congruent findings might be the similarity among study participants in terms of socio-economic background, professional qualifications, and the context of the health system. However, the motivational status in our study was higher than the findings in Jimma University Specialized Hospital (Ethiopia) and Franko Division Hospital (Cameroon), where the motivational statuses were reported as 25.1 and 42.7%, respectively (14, 23). This discrepancy could be attributed to differences in the study settings, as our study included both public and private hospitals, whereas previous studies were conducted solely in public hospitals. Additionally, the motivational status in this study was lower than that reported in other findings from West Shewa zone (Central Ethiopia) and Gaza Hospital (Europe), where motivational statuses of 63.63 and 66.2% were reported, respectively (20, 47).This disparity might be attributed to differences in the study period, settings, and design. In our study, the motivational status of health workers in private hospitals was significantly higher than in public hospitals (48, 49). This finding is consistent with previous research conducted in Bahir Dar and Hawassa Cities. The possible explanation for this difference might be due to disparities in goals, resources, and incentives between the facilities.

Health workers who had good collegial relationships were 1.61 times more likely to be motivated than those who had poor relationships. This finding is congruently similar to another study conducted in Jimma Comprehensive Specialized Hospital (14). It confirms the fact that having positive relationships with colleagues and staff in the workplace inspires individual workers and contributes to their motivation. Additionally, it was revealed that health workers who were satisfied in their job were 1.49 times more likely to be motivated compared to their counterparts. This finding was supported by previous research conducted in Jimma and West Arsi, Ethiopia (14, 39). The explanation for this finding could be that employees who are satisfied with their job tend to be more motivated to be productive in their tasks. Another significant predictor of health workers’ motivational status in our study was the presence of a favorable working environment. Health workers who worked in a favorable climate were 1.56 times more likely to be motivated than those who worked in an unfavorable climate. Similar findings were reported in other studies conducted in Gedeo zone, Ethiopia, and India (21, 50). This finding aligns with Herzberg’s two-factor theory. It might be due to the fact that less stressful working climate might favor motivation. Interestingly, more qualified health workers were found to be less motivated than those with lower educational qualifications. This finding aligns with studies conducted in Saudi Arabia, Europe, and Jimma town, Ethiopia (38, 45, 51). The possible explanation for this finding might be due to an increased expectation from educated health workers regarding the incentive packages, leading and managing opportunities. The unmet condition on these expectations might pose them less motivated.

## Study strengths and limitations

Considering health workers both working at public and private hospitals is strength of our study which the previous studies did not touch. However, it was not done without limitations. As a cross-sectional study design was employed, establishing a true causal-effect relationship was limited. Data collection during working hours might have caused respondents to be less focused due to work overload. Our study did not incorporate a qualitative approach.

## Conclusion and recommendations

The overall motivation of health workers in Bahir Dar City public and private hospitals is 56.3% which is low; challenges may be posed to maintain a regulated health workforce within the health system. Our comparison analysis revealed that motivation among health professionals was significantly higher in private hospitals than public hospitals. The overall motivational status was statistically predicted by job satisfaction, working climate, collegial relationships, educational qualification, and hospital ownership factors. Furthermore, improved compensation and staff relationships were found to decrease employee dissatisfaction, while better recognition enhanced job satisfaction, particularly in private hospitals than public hospitals. Therefore, public hospitals should promptly implement both intrinsic and extrinsic motivational strategies. Hospital administers of both sectors should focus on strategies of human resource management to increase motivated health workers through addressing financial, professional and social factors.

## Data Availability

The original contributions presented in the study are included in the article/supplementary material, further inquiries can be directed to the corresponding author.
